# Efficacy and Safety of Dapagliflozin versus Liraglutide in Patients with Overweight or Obesity and Type 2 Diabetes Mellitus: A Randomised Controlled Clinical Trial in Tianjin, China

**DOI:** 10.1155/2022/4126995

**Published:** 2022-08-13

**Authors:** Hao Zhaohu, Huang Xiao, Shao Hailin, He Feng

**Affiliations:** ^1^Department of Metabolic Disease Management Center, Tianjin 4th Central Hospital, 300140 Tianjin, China; ^2^NHC Key Laboratory of Hormones and Development (Tianjin Medical University), Tianjin Key Laboratory of Metabolic Diseases, Tianjin Medical University Chu Hsien-I Memorial Hospital & Tianjin Institute of Endocrinology, Tianjin 300134, China; ^3^Department of Cardiology, Tianjin 4th Central Hospital, 300140 Tianjin, China

## Abstract

**Objective:**

We aimed to clarify the efficacy of dapagliflozin versus liraglutide in patients with overweight or obesity and type 2 diabetes mellitus (T2DM) at the beginning of the coronavirus disease 2019 (COVID-19) pandemic.

**Methods:**

T2DM patients with overweight or obesity who visited the Metabolic Disease Management Center at Tianjin Fourth Central Hospital from October 2019 to January 2020 were recruited and randomised to receive dapagliflozin or liraglutide for 24 weeks. Changes in blood glucose and lipid levels, blood pressure, and body weight, as well as the occurrence of hypoglycaemia and other adverse events, were compared.

**Results:**

309 patients completed the study (143 in liraglutide group and 166 in dapagliflozin group). After 24 weeks, HbA1c, fasting blood glucose (FPG), and 2 h postprandial blood glucose (2hPG) levels significantly decreased from 8.80% ± 1.41% to 7.02% ± 1.05%, 10.41 ± 3.13 to 7.59 ± 2.16 mmol/L, and 17.90 ± 4.39 to 10.12 ± 2.47 mmol/L, respectively, in the dapagliflozin group, and from 8.92% ± 1.49% to 6.78% ± 1.00%, 10.04 ± 2.99 to 7.20 ± 1.63 mmol/L, and 17.30 ± 4.39 to 10.13 ± 4.15 mmol/L, respectively, in the liraglutide group. Changes in HbA1c, FPG, and 2hPG levels between groups were not significantly different. Systolic blood pressure (SBP) and low-density lipoprotein cholesterol (LDL-C) level significantly decreased from 144.1 ± 19.1 to 139.7 ± 16.2 mmHg (*p* = 0.001) and from 3.21 ± 0.94 to 2.98 ± 0.89 mmol/L (*p* = 0.014), respectively, in the dapagliflozin group. After COVID-19 outbreak, the number of patients taking sleep-promoting drugs increased from 4.9% to 9.4% (*p* = 0.029).

**Conclusions:**

Liraglutide and dapagliflozin had strong hypoglycaemic effects in patients with overweight or obesity and T2DM at the beginning of the COVID-19 pandemic. Dapagliflozin may be beneficial in improving SBP and LDL-C levels; however, further research is warranted.

## 1. Introduction

Type 2 diabetes mellitus (T2DM) is a common chronic metabolic disease with an increasing global prevalence. According to WHO criteria, the number of patients with diabetes mellitus (DM) in China has rapidly increased to 11.2%, and the number of patients with T2DM is the highest in the world [1]. The number of global deaths due to diabetes in 2000 was estimated at 2.9 million, accounting for 5.2% of all deaths [2]. T2DM increases all-cause mortality, including cardiovascular, stroke-associated, and ischemic heart disease mortalities [3, 4]. Obesity is one of the major risk factors for developing T2DM, and its high global incidence promotes increases in cardiovascular morbidity and mortality rates [5]. There is low compliance with blood glucose monitoring and subsequent control in China, and data from the China National HbA1c Surveillance System showed that the HbA1c (<7%) compliance rate in China is only 27.7% [6].

Metformin is a safe, low-cost, widely used, hypoglycaemic drug that has an outstanding ability to decrease plasma glucose levels and has been employed for over 60 years to treat early stages of T2DM [7]. Additionally, metformin has other beneficial effects; for example, it is a candidate drug for reducing the risk of amiodarone-induced hyperthyroidism and interstitial lung disease [8]. However, fewer patients than expected receive metformin as first-line monotherapy because of secondary failure [9]. Therefore, it is important to select hypoglycaemic drugs that can be used in combination with metformin to benefit patients with T2DM. T2DM is associated with a substantially increased risk of death in Chinese adults, especially relating to cardiovascular disease, and almost 50% of such deaths are caused by stroke [10].

Sodium-glucose transporter-2 inhibitors (SGLT2is) [11] and glucagon-like peptide-1 receptor agonists (GLP-1RAs) [12] have been shown to have multiple cardiovascular and renal benefits in patients with diabetes. Current studies also suggest that these benefits apply to T2DM patients with multiple comorbidities, including chronic kidney disease [13] and heart failure with reduced ejection fraction [14].

Coronavirus disease 2019 (COVID-19) threatened the world as a new public health crisis following its emergence in Wuhan, Hubei Province, China, in December 2019 [15]. Cities in China, including Tianjin, took strict epidemic prevention measures. Few studies focused on the efficacy and safety of prescribing a combination of SGLT2i and GLP-1RA to patients with overweight or obesity and T2DM with poor blood glucose control who are using metformin in China during the COVID-19 pandemic. This study was aimed at comparing the efficacy and safety of dapagliflozin and liraglutide in patients with overweight or obesity and T2DM during this period.

## 2. Materials and Methods

### 2.1. Participants

Patients with T2DM who visited the Metabolic Disease Management Center (MMC) at Tianjin Fourth Central Hospital from October 2019 to January 2020 were recruited for the study. The study inclusion criteria were as follows: (a) age ≥ 18 years, (b) body mass index (BMI) ≥ 24 kg/m^2^ [16], (c) stable dose of metformin (≥1500 mg/d) alone or in combination with premixed insulin for ≥8 weeks, and (d) HbA1c level ≥ 7.0%.

The exclusion criteria were as follows: (a) type 1 and other special types of diabetes such as gestational diabetes, (b) severe mental illness and unclear consciousness, (c) active tuberculosis and other infectious diseases, and (d) high risk for volume depletion, hypotension, and/or electrolyte imbalances (in the opinion of the investigator). Laboratory exclusion criteria included haemoglobin < 120 g/L (male), <110 g/L (female), or thyroid-stimulating hormone levels outside the central laboratory normal range.

Enrolled patients voluntarily withdrew from the study during the observation period. Written informed consent was obtained from all participants.

### 2.2. Study Design

This was a single-centre, randomised, parallel, controlled clinical observational study that lasted for 24 weeks. According to the random number table and time sequence of patient enrolment, the researchers divided the patients meeting enrolment conditions into dapagliflozin and liraglutide treatment groups at a ratio of 1 : 1. Dapagliflozin was initiated at 5 mg and titrated up to 10 mg by the second week unless (in the opinion of the investigator) the patient was unable to tolerate titration to 10 mg, in which case the dose was maintained at 5 mg. The liraglutide group patients were subcutaneously injected 0.6 mg/d at the beginning, and this dose increased to 1.2 mg/d by the second week. If intolerance occurred during the process, the dose was adjusted to 0.6 mg/d.

According to the MMC system, we collected the following information: name, sex, age, contact information, smoking/drinking history, family history of diabetes, history of hypertension, coronary heart disease (CHD), and diabetes course. Blood glucose, lipid, and HbA1c levels; body weight; and blood pressure were monitored and recorded during the observation period. The body weight of patients and systolic and diastolic blood pressure levels were recorded at baseline, week 12, and week 24. The blood index monitoring plan was as follows: (1) at baseline: fasting peripheral blood glucose (FPG), 2 h postprandial peripheral blood glucose (2hPG), HbA1c, alanine aminotransferase, aspartate aminotransferase, blood urea nitrogen, serum creatinine, haemoglobin, triglyceride (Tg), low-density lipoprotein cholesterol (LDL-C), fasting venous blood glucose, and serum insulin levels; (2) week 12: FPG, 2hPG, and HbA1c levels; and (3) week 24: FPG, 2hPG, HbA1c, Tg, and LDL-C levels. Nurses provided education regarding diet and exercise, blood glucose and blood pressure monitoring, and liraglutide injection techniques, as well as the identification and treatment of adverse events (AEs), including hypoglycaemia. AEs were recorded and treated throughout the study.

The outbreak of COVID-19 in December 2019 caused enormous disruption to the daily routines of the global community [17]. Various measures [18] were applied to prevent and control disease progression and minimise the impact of the pandemic in China. Tianjin reported its first case of COVID-19 in January 2020, and strict measures were taken to limit the outdoor activities of the residents from January to April 2020. Such measures altered implementation of the usual follow-up procedures. Tianjin Fourth Central Hospital provided door-to-door drug delivery services for patients to avoid drug disconnection. The researchers contacted the patients by phone and WeChat every two weeks to determine any difficulties and provide the patients with home exercise programs. The lives of Tianjin residents gradually returned to normal in April 2020, but COVID-19 became a global pandemic that affected the health and well-being of most people. In addition to the physical, economic, and social impacts, the psychological impacts of this pandemic have been increasingly reported in scientific literature [19]. Therefore, changes in sleep quality before and after the epidemic were also assessed using questionnaires in this study.

The clinical study protocol was approved by the Institutional Review Board (IRB) of Tianjin 4^th^ Central Hospital, and all steps were conducted in accordance with the principles of the World Medical Association Declaration of Helsinki (trial registration code: ChiCTR1800019864). The IRB approved the collection and use of patient records according to the regulations for clinical trials in humans (IRB approval No. 2018-SZXLL066).

### 2.3. Study Evaluations

The primary objectives of this study were as follows: (a) after 24 weeks, to assess the effect of the addition of dapagliflozin compared to the addition of liraglutide on HbA1c level, and (b) over 24 weeks, to assess the overall safety and tolerability of dapagliflozin compared to liraglutide.

The secondary objectives were as follows: to assess 2 h incremental postprandial glucose excursion (PPGE), FPG, 2hPG, and the proportion of subjects with an HbA1c goal < 7.0% after 24 weeks of treatment. Changes in Tg and LDL-C levels, body weight, and blood pressure from baseline were compared between the two groups. Venous blood samples were collected in EDTA tubes from fasting patients in the morning. HbA1c levels were determined using affinity chromatography in a hospital standard laboratory (Tosoh Corporation, Japan).

### 2.4. Indicators and Evaluation Criteria

The following evaluation criteria were employed: (1) T2DM: the diagnosis of T2DM was based on the 2020 Chinese diabetes treatment guidelines: FPG level ≥ 7.0 mmol/L (fasting was defined as no caloric intake for at least 8 h) or 2 h plasma glucose level ≥ 11.1 mmol/L [20]. (2) BMI: an Omron infrared height and weight meter was used to automatically measure the height and weight. Body weight was measured using the same scale on an empty stomach at the MMC clinic in the morning. BMI was calculated as body weight (kg)/height squared (m^2^). Patients with BMI (≥24 and <28 kg/m^2^) were considered overweight, and those with BMI ≥ 28 kg/m^2^ were considered obese [16]. (3) Homeostatic Model Assessment Insulin Resistance (HOMA-IR) index [21]: FPG level (mmol/L) × fasting plasma insulin level (mIU/L)/22.5. (4) PPGE: calculated from peripheral blood glucose level before and after breakfast in the present study. (5) The standards of high quality rate (HQR) [22] included (a) no hypoglycaemia, (b) weight gain < 2%, and (c) HbA1c level < 7% at week 24.

A sleep quality questionnaire was used in this study. Sleep quality was considered poor if it affected normal work during the day. Patients were also asked whether they took medication to promote sleep.

The following safety indicators were evaluated: (1) hypoglycaemia: hypoglycaemia was diagnosed at a blood glucose level lower than 3.9 mmol/L [20]. In this study, hypoglycaemia diagnosis was based on patient self-reports and blood glucose monitoring [23]. Severe hypoglycaemic events were considered when disturbance of consciousness or symptoms that could not be self-managed occurred. (2) Identification of serious adverse events (SAEs): SAEs included myocardial infarction, cardiac surgery or revascularisation, unstable angina pectoris, congestive heart failure, transient ischemic attack, severe cerebrovascular disease, severe hypoglycaemic events, hypertonic coma, and ketoacidosis. All events were reported to the investigators. To ensure compliance with the protocol definitions, rigorous measures were implemented to ensure data quality, including source data verification for reported outcomes and safety events and a thorough review of events.

### 2.5. Efficacy and Safety Endpoints

Glycaemic efficacy endpoints were changes from baseline in HbA1c, 2 h incremental PPGE, 2hPG, and FPG levels and the proportion of patients who achieved an HbA1c goal of <7% at week 24. Safety endpoints included AEs, hypoglycaemia, or urinary tract infection.

Other indicators included changes in body weight, blood lipid levels, and blood pressure from baseline to week 24.

### 2.6. Statistical Analyses

In this study, the GPower software was used to estimate the required sample size and assist in evaluating the effect size and statistical efficacy. The Statistical Program for Social Sciences 26.0 software (SPSS, Inc., Chicago, IL, USA) was used for data collection and analysis. The Kolmogorov–Smirnov normal test was performed on the measurement data, the mean ± standard deviation was used to describe variables conforming to a normal distribution, and percentage (%) was used for counting data. An independent sample *t*-test was used to compare measurement data between the two groups, and a paired sample *t*-test was used to compare the groups before and after treatment. The chi-square test was used to compare the observed data. All statistical tests were performed using bilateral tests with an alpha of 0.05.

## 3. Results

### 3.1. Patient Disposition and Characteristics and Dapagliflozin/Liraglutide Doses

**Figure 1:**
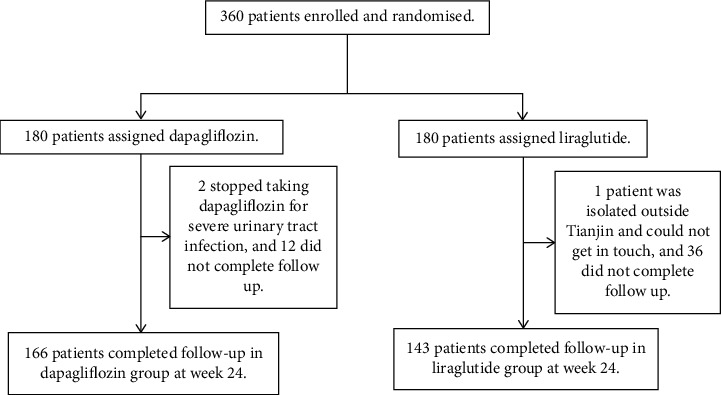
The study flow chart.

A total of 360 patients were eligible for enrolment and volunteered to participate in the study from October 2019 to January 2020. There were 180 participants in each group, but only a total of 309 patients completed the study (Figure 1). There were 166 and 143 patients in the dapagliflozin and liraglutide groups, respectively, at week 24. The mean age of the participants was 51.8 ± 11.2 years, and 190 patients (61.5%) were male. Patients had a mean BMI of 29.86 ± 4.25 kg/m^2^, mean HbA1c level of 8.85% ± 1.44%, and average T2DM duration of 6.3 ± 5.7 years. The baseline demographics and clinical parameters of the two groups are shown in Table 1. Moreover, 160 patients (96.3%) in the dapagliflozin group were administered 10 mg of the drug per day, and 130 patients (90.9%) in liraglutide group were administered 1.2 mg of the drug per day. None of the patients had COVID-19.

### 3.2. Efficacy

After 24 weeks, significant decreases in HbA1c (liraglutide group: −2.14%, *t* = 14.180, *p* ≤ 0.001; dapagliflozin group: −1.78%, *t* = 14.983, *p* ≤ 0.001), FPG (liraglutide group: −2.95 mmol/L, *t* = 10.388, *p* ≤ 0.001; dapagliflozin group: −2.83, *t* = 9.78, *p* ≤ 0.001), and 2hPG (liraglutide group: −7.47 mmol/L, *t* = 8.969, *p* ≤ 0.001; dapagliflozin group: −6.36 mmol/L, *t* = 8.464, *p* ≤ 0.001) levels occurred in both groups (Table 2). There were no significant differences in the HbA1c, FPG, and 2hPG levels between the two groups.

**Figure 2:**
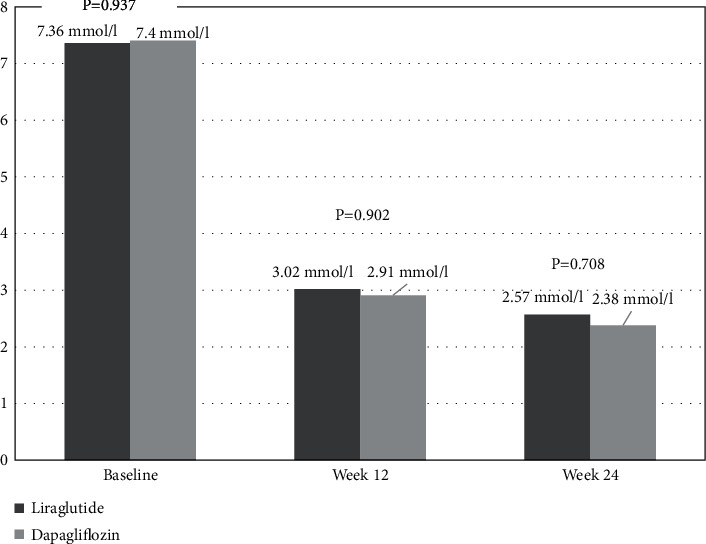
Comparison of PPGE between the two groups at week 12 and week 24. Abbreviations: PPGE: 2-hour incremental postprandial glucose excursion.

Compared with baseline data (Figure 2), there was no significant difference in PPGE between the liraglutide and dapagliflozin groups at week 12 (3.02 ± 3.57 mmol/L vs. 2.91 ± 3.32 mmol/L, *t* = 0.124, *p* = 0.902) and week 24 (2.57 ± 2.33 mmol/L vs. 2.38 ± 2.62 mmol/L, *t* = 0.376, *p* = 0.708).

**Figure 3:**
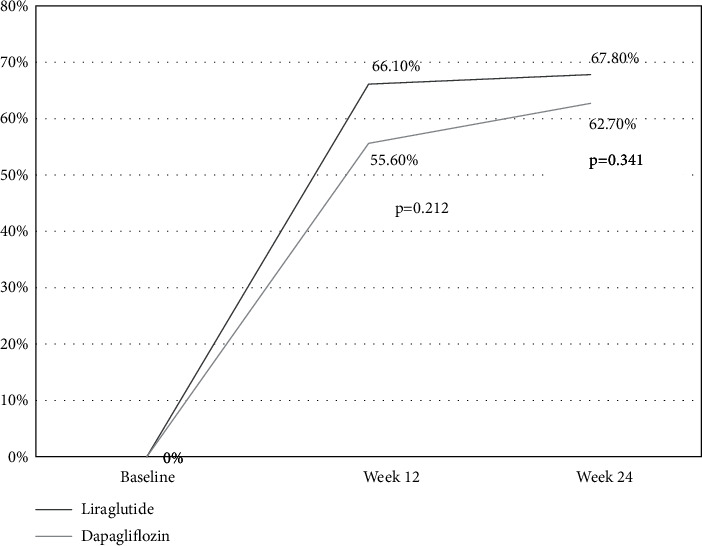
Comparison of HbA1c compliance rate (<7.0%) between the two groups.

There was no significant difference in the compliance rate of HbA1c (<7.0%) between the liraglutide and dapagliflozin groups (Figure 3) at weeks 12 (66.1% vs. 55.6%, *χ*^2^ = 1.558, *p* = 0.212) and 24 (67.8% vs. 62.7%, *χ*^2^ = 0.907, *p* = 0.341). Additionally, there was no significant difference in the HQR between the liraglutide and dapagliflozin groups at week 24 (44.6% vs. 41.3%, *χ*^2^ = 0.319, *p* = 0.572).

### 3.3. Changes in Other Metabolic Indicators (Body Weight, SBP, DBP, and TG, and LDL-C Levels)

The changes in body weight, SBP, DBP, and TG and LDL-C levels in the two groups after 24 weeks of treatment are shown in Table 3. There were no significant changes in TG and LDL-C levels, body weight, or blood pressure in the liraglutide group after 24 weeks of treatment. However, significant decreases were observed in the SBP (−5.28 (−8.43–−2.12), *t* = 3.306, *p* = 0.001) and LDL-C levels (−0.24 (−0.42–−0.05), *t* = 2.512, *p* = 0.014) in the liraglutide group. There were no significant changes in TG level, body weight, and DBP.

### 3.4. Safety Endpoints

During the 24-week observation period, 50 patients experienced mild hypoglycaemia events (21 (14.7%) and 29 (17.5%) patients in the liraglutide and dapagliflozin groups, respectively). No significant differences between the two groups were noted (*χ*^2^ = 0.439, *p* = 0.508). A total of 20 patients had urinary tract infection, including 5 and 15 in the liraglutide and dapagliflozin groups, respectively, and 2 of them stopped treatment with dapagliflozin due to a severe urinary tract infection. There was no significant difference between the two groups (*χ*^2^ = 3.788, *p* = 0.052).

During the observation period, nine patients experienced chest tightness, feelings of suffocation, and other discomfort. Five patients were relieved of their symptoms after psychological counselling. Four patients were diagnosed with angina pectoris and were further hospitalised (two in each group). No serious AEs, such as severe hypoglycaemia, heart failure, or myocardial infarction, were noted in either group.

### 3.5. Sleep Quality

Following the start of the COVID-19 outbreak, the proportion of patients with poor sleep quality significantly increased from 27.5% to 41.7% (*χ*^2^ = 13.839, *p* ≤ 0.001). The number of patients taking sleep-promoting drugs significantly increased from 15 (4.9%) to 29 (9.4%) (*χ*^2^ = 4.796, *p* = 0.029).

## 4. Discussion

The results showed that both liraglutide and dapagliflozin significantly reduced HbA1c and fasting and postprandial blood glucose levels in patients with overweight or obesity and T2DM. This study showed that even in the initial stage of the COVID-19 outbreak, when the lifestyle of patients was seriously affected, HbA1c levels were consistently reduced. Few current studies have compared these drugs, but a meta-analysis found that compared to other antidiabetic drugs (including SGLT2I), GLP-1RAs, including liraglutide and dulaglutide, provided better hypoglycaemic effects [24]. A retrospective multicentre study conducted at 46 diabetes specialist outpatient clinics in Italy compared the hypoglycaemic efficacy of dapagliflozin (10 mg/d) and liraglutide (1.2 mg/d) in real-world patients between 2015 and 2016, and the results showed similar endpoints [25]. A study in China from 2017 to 2018 found that after a 24-week treatment period, higher reductions in HbA1c level were observed with dapagliflozin (10 mg/d) than with liraglutide (1.8 mg/d) [26]. However, the results of our study showed no differences in the changes in FPG, 2hPG, PPGE, or HbA1c levels between the two groups before and after 24 weeks of treatment.

Liraglutide, a GLP-1RA, mediates several positive effects, including lowering glucose dependence and reducing appetite and body weight; furthermore, it provides antiatherosclerosis, neural protection, natriuresis, and bone osteogenesis benefits [27]. Dapagliflozin is a new oral hypoglycaemic drug that increases urinary glucose excretion by inhibiting glucose reabsorption in proximal renal tubules. SGLT2is can reduce HbA1c levels without increasing the risk of hypoglycaemia, induce weight loss, and improve various metabolic parameters, including blood pressure, lipid profile, and hyperuricemia [28].

Obesity is one of the main risk factors for T2DM, and both constitute a major global health crisis [29]. Weight management is becoming increasingly important for the diagnosis and treatment of T2DM [30]. Liraglutide and dapagliflozin are potent hypoglycaemic drugs that have been widely recommended in China for managing the weight of patients with T2DM [20]. Treatment with liraglutide was found to attenuate cardiometabolic dysregulation and improve cardiac function, while dapagliflozin treatment improved glucose handling but had only a mild effect on an animal model of heart failure with preserved ejection fraction [31].

However, in the present study, there were no significant changes in body weight between the two groups before and after the 24-week treatment period. Body weight is regulated by the interaction of a number of processes, including homoeostatic, environmental, and behavioural factors. Interventions based on lifestyle modifications are integral to the management of body weight [32]. It is possible that the sudden outbreak of the epidemic and local policies had a certain impact on the activities and emotions of patients and may even have affected thyroid function [33]. In our study, we also found that the quality of sleep had deteriorated in patients during the outbreak. Studies have suggested a potential causal relationship between poor sleep and rapid weight gain, which may be related to the effects of sleep on dietary intake or physical activity [34].

The SBP of patients in the dapagliflozin group decreased significantly before and after the 24-week treatment period compared to the liraglutide group. Dapagliflozin at a dose of 10 mg has been found to induce a modest reduction in blood pressure compared with placebo in patients with diabetes with a low risk of orthostatic reactions, regardless of baseline blood pressure, and without increasing the heart rate [35]. The associated mechanisms include osmotic diuresis, bulbar balance, and weight loss [28]. In addition, recent studies have suggested that dapagliflozin has an important effect on lipid metabolism. It is also known that dapagliflozin can reduce blood TG [36] and blood uric acid [37] levels and increase the level of high-density lipoprotein cholesterol [38]. One study showed that LDL-C level increases significantly in patients after SGLT-2i treatment [39], whereas the levels of small and dense LDL decrease significantly, which is beneficial for atherosclerotic diseases [36]. In contrast with previous studies, our study showed a significant reduction in LDL-C levels in patients treated with dapagliflozin. This result could be related to interfering factors, such as the use of lipid-lowering drugs, as well as the impact of COVID-19. In patients receiving metformin-based background therapy who have an increased cardiovascular risk, specific GLP-1RAs and SGLT-2is have demonstrated favourable effects on certain cardiovascular outcomes [40]. However, studies have also shown that the effects of dapagliflozin and liraglutide on bone material properties are not identical and are not only mediated by low blood glucose levels [41]. Therefore, other effects of these two new hypoglycaemic drugs need to be confirmed in further studies.

Both treatments were well-tolerated. In the dapagliflozin group, two patients left the study after contracting urinary tract infections. SGLT2is increased the overall risk of genital system infection but did not increase the risk of other safety events, such as amputation, fracture, acute renal injury, and hyperkalaemia. The safety outcomes of the study were consistent with those of previous studies [42].

## 5. Conclusions and Limitations

Even under the impact of COVID-19, liraglutide and dapagliflozin have strong hypoglycaemic effects in patients with overweight or obesity and T2DM. Compared to liraglutide, dapagliflozin may greatly improve blood pressure and blood lipid levels. Further research is needed to confirm this assumption. This study, however, has many limitations. The study is a single-centre study, and the enrolled patients were limited to the northern area of Tianjin. Since liraglutide is an injectable drug and dapagliflozin is an oral hypoglycaemic drug, the study was not double-blinded, and the conclusions need further verification.

## Figures and Tables

**Table 1:** Baseline demographic, anthropometric, and disease characteristics between the study groups.

	Liraglutide *n* = 143	Dapagliflozin *n* = 166	*χ* ^2^/*t* value	*p* value
Age, years	51.9 ± 11.1	51.8 ± 11.4	0.104^∗^	0.917
Male, *n* (%)	83 (58.0%)	107 (64.5%)	1.335^#^	0.248
Urban residence patients, *n* (%)	103 (72.0%)	114 (68.7%)	0.413^#^	0.520
Smoking history, *n* (%)	60 (42.0%)	73 (44.0%)	0.128^#^	0.721
Drinking history, *n* (%)	22 (15.4%)	28 (16.9%)	0.125^#^	0.724
DM family history, *n* (%)	106 (74.1%)	103 (62.0%)	5.119^#^	0.024
Hypertension, *n* (%)	108 (75.5%)	126 (75.9%)	0.006^#^	0.938
CHD, *n* (%)	29 (20.3%)	26 (15.7%)	1.119^#^	0.290
T2DM duration (years)	6.5 ± 5.8	6.1 ± 5.5	0.504^∗^	0.615
SBP (mmHg)	143.9 ± 22.5	144.1 ± 19.1	-0.088^∗^	0.930
DBP (mmHg)	83.5 ± 13.0	85.5 ± 11.9	-1.442^∗^	0.150
Body weight (kg)	85.5 ± 14.5	84.3 ± 15.0	0.693^∗^	0.489
BMI (kg/m^2^)	30.2 ± 4.3	29.6 ± 4.2	1.212^∗^	0.226
TSH	2.53 ± 2.39	2.12 ± 1.28	1.104^∗^	0.272
FT4	14.31 ± 2.51	13.53 ± 2.04	1.737^∗^	0.085
Tg	2.34 ± 1.50	2.62 ± 1.88	-1.248^∗^	0.213
LDL-C	3.48 ± 1.06	3.21 ± 0.94	2.090^∗^	0.038
BUN	5.34 ± 1.51	5.39 ± 1.52	-0.266^∗^	0.791
Scr	60.2 ± 14.8	65.6 ± 21.0	-2.261^∗^	0.025
ALT	32.6 ± 21.1	39.6 ± 33.5	-1.878^∗^	0.062
AST	25.2 ± 12.9	27.4 ± 19.1	-1.028^∗^	0.305
HbA1c (%)	8.92 ± 1.49	8.80 ± 1.41	0.773^∗^	0.440
FPG (mmol/l)	10.04 ± 2.99	10.41 ± 3.13	-1.048^∗^	0.295
2hPG (mmol/l)	17.30 ± 4.39	17.90 ± 4.39	-1.118^∗^	0.264
PPGE (mmol/l)	7.36 ± 4.67	7.40 ± 4.36	-0.079^∗^	0.937
HOMA-IR	5.83 ± 3.94	6.99 ± 7.36	-1.629^∗^	0.124
Background medication, *n* (%)				
Metformin+SU	40 (28.0%)	51 (30.7%)	0.280^#^	0.597
ACEI/ARB	80 (55.9%)	95 (56.5%)	0.011^#^	0.915
Aspirin	26 (18.2%)	29 (17.3%)	0.045^#^	0.832
Statins	51 (35.7%)	50 (29.8%)	1.227^#^	0.268

Abbreviations: DM: diabetes mellitus; CHD: coronary atherosclerotic heart disease; T2DM: type 2 diabetes mellitus; SBP: systolic blood pressure; DBP: diastolic blood pressure; BMI: body mass index; Tg: triglycerides; LDL-C: low-density lipoprotein cholesterol; BUN: blood urea nitrogen; Scr: serum creatinine; ALT: alanine aminotransferase; AST: aspartate aminotransferase; FPG: fasting peripheral blood glucose; 2hPG: 2 h postprandial peripheral blood glucose; PPGE: 2-hour incremental postprandial glucose excursion; SU: sulfonylurea; HOMA-IR: homeostatic model assessment insulin resistance index; ^∗^*t* value; ^#^*χ*^2^ value.

**Table 2:** Efficacy endpoints (HbA1c, FPG, and 2hPG) at week 24.

Parameter	Liraglutide (*n* = 143)	Dapagliflozin (*n* = 166)
HbA1c (%)		
Baseline	8.92 ± 1.49	8.80 ± 1.41
Week 24	6.78 ± 1.00	7.02 ± 1.05
Change from baseline^a^	-2.14^c^ (-2.45~-1.85)	-1.78^c^ (-2.01~-1.55)
Change vs. dapagliflozin^b^	*t* = 1.910 *p* = 0.057	
FPG (mmol/l)		
Baseline	10.04 ± 2.99	10.41 ± 3.13
Week 24	7.20 ± 1.63	7.59 ± 2.16
Change from baseline^a^	-2.95^c^ (-3.52~-2.39)	-2.83^c^ (-3.40~-2.26)
Change vs. dapagliflozin^b^	*t* = 0.304 *p* = 0.761	
2hPG (mmol/l)		
Baseline	17.30 ± 4.39	17.90 ± 4.39
Week 24	10.13 ± 4.15	10.12 ± 2.47
Change from baseline^a^	-7.47^c^ (-9.14~-5.79)	-6.36^c^ (-7.87~-4.85)
Change vs. dapagliflozin^b^	*t* = 0.988 *p* = 0.326	

Abbreviations: FPG: fasting peripheral blood glucose; 2hPG: 2-hour postprandial peripheral blood glucose. ^a^*t* test of paired samples before and after treatment, *p* = 0.000. ^b^Independent sample *t* test of change value between two groups. ^c^Mean change (95% confidence interval).

**Table 3:** Changes in other metabolic indicators (body weight, Tg, LDL-C, DBP, and SBP) at week 24.

Parameter	Liraglutide *n* = 143	Dapagliflozin *n* = 166
Body weight (kg)		
Baseline	85.5 ± 14.5	84.3 ± 15.0
Week 24	85.5 ± 16.4	82.7 ± 12.9
Change from baseline^a^	-0.08 (-1.78~1.94)	-0.61 (-1.23~0.01)
*t* value	-0.086	1.939
*p* value	0.931	0.055
SBP (mmHg)		
Baseline	143.9 ± 22.5	144.1 ± 19.1
Week 24	140.6 ± 23.7	139.7 ± 16.2
Change from baseline^a^	-3.98 (-8.87~0.92)	-5.28 (-8.43~-2.12)
*t* value	1.608	3.306
*p* value	0.111	0.001
DBP (mmHg)		
Baseline	83.5 ± 13.0	85.5 ± 11.9
Week 24	84.0 ± 12.7	84.6 ± 10.6
Change from baseline^a^	0.38 (2.92~-1.28)	-1.12 (-2.91~0.67)
*t* value	-0.296	1.233
*p* value	0.767	0.220
Tg (mmol/l)		
Baseline	2.34 ± 1.50	2.62 ± 1.88
Week 24	2.27 ± 1.91	2.46 ± 1.85
Change from baseline^a^	-0.24 (-0.49~0.01)	-0.15 (-0.49~0.18)
*t* value	1.974	0.907
*p* value	0.053	0.367
LDL-C (mmol/l)		
Baseline	3.48 ± 1.06	3.21 ± 0.94
Week 24	3.29 ± 1.13	2.98 ± 0.89
Change from baseline^a^	-0.24 (-0.49~0.02)	-0.24 (-0.42~-0.05)
*t* value	1.837	2.512
*p* value	0.071	0.014

Abbreviations: SBP: systolic blood pressure; DBP: diastolic blood pressure; Tg: triglycerides; LDL-C: low-density lipoprotein cholesterol. ^a^Mean change (95% confidence interval).

## Data Availability

Data are available upon reasonable request by email.
